# “Mom Let’s Go to the Dentist!” Preliminary Feasibility of a Tailored Dental Intervention for Children with Autism Spectrum Disorder in the Italian Public Health Service

**DOI:** 10.3390/brainsci10070444

**Published:** 2020-07-12

**Authors:** Antonio Narzisi, Mariasole Bondioli, Francesca Pardossi, Lucia Billeci, Maria Claudia Buzzi, Marina Buzzi, Martina Pinzino, Caterina Senette, Valentina Semucci, Alessandro Tonacci, Fabio Uscidda, Benedetta Vagelli, Maria Rita Giuca, Susanna Pelagatti

**Affiliations:** 1 IRCCS Stella Maris Foundation, 56018 Pisa (Calambrone), Italy; antonio.narzisi@fsm.unipi.it; 2 Department of Informatics, University of Pisa, 56127 Pisa, Italy; mariasole.bondioli@gmail.com (M.B.); fabio.usc@gmail.com (F.U.); 3 Unit of Odontostomatology and Oral Surgery, University Hospital of Pisa, 56126 Pisa Italy; f.pardossi@yahoo.it (F.P.); mariarita.giuca@med.unipi.it (M.R.G.); 4 Institute of Clinical Physiology, National Research Council of Italy, (IFC-CNR), 56124 Pisa, Italy; lucia.billeci@ifc.cnr.it (L.B.); atonacci@ifc.cnr.it (A.T.); 5 Institute of Informatics and Telematics, National Research Council of Italy, (IIT-CNR), 56125 Pisa, Italy; claudia.buzzi@iit.cnr.it (M.C.B.); marina.buzzi@iit.cnr.it (M.B.); caterina.senette@iit.cnr.it (C.S.); 6 Institute of Neuroscience, National Research Council of Italy of Italy, (IN-CNR), 56125 Pisa, Italy; martinapinzino@outlook.it; 7 UFSMIA, Zona Livorno, Azienda USL Toscana Nord Ovest, 57124 Livorno, Italy; valentina.semucci@uslnordovest.toscana.it; 8 UFSMIA, Zona Pisana, Azienda USL Toscana Nord Ovest, 56121 Pisa, Italy; benedetta.vagelli@uslnordovest.toscana.it; 9 Department of Surgical, Medical and Molecular Pathology and Critical Care Medicine, University of Pisa, 56126 Pisa, Italy; 10 Autismo Pisa APS, Autism Parents Association, 56100 Pisa, Italy

**Keywords:** autism, dental care, oral health, medical procedures, ICT, wearable sensors

## Abstract

Children with autism spectrum disorder (ASD) show worse oral health than their peers. Their access to health services is, at present, inadequate: few high-quality interventions have been designed and implemented to improve their care procedures so far. The purpose of this study is to describe an experience of dental care supported by Information and Communication Technologies (ICT), for children with ASD in a public health service. In our study, 59 children (mean age 9.9 years; SD = 5.43) participated in the MyDentist project. It integrates classic dental care techniques with new practices for desensitization and fear control, delivered through an enhanced customized ICT-based intervention aiming at familiarizing the child with ASD with the medical setting and procedures. Two questionnaires were filled out by parents to describe the acceptability of the MyDentist experience for their children. Significant results were shown from T0 (before initiating MyDentist) to T1 (after 6 months of the MyDentist experience) regarding improved oral hygiene and cooperation during dental treatments. Families positively assessed the use of ICT support. In conclusion, the project demonstrated acceptability by parents, suggesting that public health dental care and prevention can be successfully implemented without resorting to costly pharmacological interventions (with potential side effects), taking better care of children’s health.

## 1. Introduction 

Autism spectrum disorder (ASD) is a severe multifactorial disorder characterized by an umbrella of specific symptoms in the areas of social communication, restricted interests, and repetitive behaviors [1]. The incidence of ASD is worldwide and recent epidemiological data estimated it to be 1/54 in United States and 1/87 in Italy [2,3]. ASD varies greatly in the severity of associated socio-communicative impairments and in the degree of cognitive and language development [4]. Although there is no specific relationship between ASD and oral disease, it is well-recognized that many individuals with ASD have much worse oral health than non-autistic people [5,6,7,8,9,10]. This may be related to barriers to dental services, sensory sensitivities and heightened levels of stress and anxiety during care; these factors may affect the level of cooperation of individuals with ASD in regard to daily hygiene routines, oral exams, and dental care [11,12,13,14,15,16,17]. The presence of comorbid self-injurious behaviors, poor oral care at home and specific dietary habits that can favor tooth decay should also be considered [17,18].

Sensory sensitivity is extremely heterogeneous in subjects with ASD [19,20]. It can include hyper-sensitivity to specific sensory input. Subjects with ASD can also experience sensory overload, becoming overwhelmed by incoming stimuli. In these situations, sights, sounds, smells, tastes, touch, balance and body awareness can feel like real physical pain [19,20].

These children can also present intellectual disabilities and significant behavioral issues [21].

These features make it difficult for dentists to assess and treat children with ASD; due to sensory sensitivity and/or intellectual disability, it may be necessary to resort to general anesthesia or sedation (conscious or deep) for dental care or invasive treatment [22,23,24].

Unfortunately, the professional training offered by most university programs does not deal specifically with people with ASD, so it is necessary to teach dental professionals how to treat them correctly [22]. Children with ASD need to be supported and guided during the visit by an interdisciplinary plan of action that takes into account their specific needs in order to promote better dental health [22,25,26]. Therefore, to increase the probability of successful dental treatment for patients with ASD, dentists should have an in-depth interdisciplinary understanding of ASD in terms of symptomatology and functional profiles [27,28]. However, as highlighted by the World Health Organization (WHO) and American Academy of Pediatric Dentistry, access to services and support for people with ASD is presently inadequate [29,30].

In order improve cooperation of patients with ASD in a dental setting, a link between the behavioral approach and the personalized use of information and communication technologies (ICT) could be efficacious. According to previous studies conducted on children with ASD, it is possible to achieve improved oral hygiene also with both Augmentative Alternative Communication (AAC) and online video modeling sessions [31,32]. The AAC encompasses the communication methods used to supplement speech for those with specific impairments in the production or comprehension of spoken language. Specifically, AAC technologies support children with ASD to (a) enhance language learning, (b) facilitate social interaction and (c) teach interaction strategies to communication partners [31]. Video modeling is a mode for teaching behaviors that uses video recording to provide a visual model of the targeted behavior or skill. Video modeling could include basic video modeling, video self-modeling, point-of-view video modeling, and video prompting [32].

This type of studies [31,32] provides preliminary support for the use of ICT-enhanced interventions to improve oral hygiene for children with autism [33,34]. Providing dental care to patients with ASD may require modifying the traditional treatment plans and settings [33,34], which include many sensory stimuli that may affect the way children behave. Therefore, it is extremely important to think about functional ways to reduce these users’ anxiety and sensory hypersensitivity in order to increase their compliance with procedures and to carry out dental care as planned. Despite the high prevalence of issues in treating people with ASD, only few randomized controlled pilot studies have been designed and implemented to improve their dental care procedures so far [17,35,36,37]. The extreme importance of oral health in children with ASD is due to the potential beneficial effects on chewing/eating, language disorder and dental traumas [30].

The aim of this paper is to describe the first results of the project MyDentist, an in-the-field experience of dental care for children with ASD in an Italian public health service, with the support of a web application that includes various digital materials (video models, social stories, photos, and games), which can be personalized for each patient.

From a clinical point of view, the two specific aims of the MyDentist project were to provide adequate dental care for subjects with ASD and to support and motivate their maintaining acceptable oral health at home. The first aim focused on increasing the patient’s collaboration when sitting in the dentist chair, considering the time needed by each specific patient when performing from the simplest to the most complex therapeutic treatment. In this way, it would be possible to reduce the use of general anesthesia, at least for those treatments that generally do not need it, thus lowering economic and psychological costs.

The second aim was choosing the most appropriate method to guide the patient during their oral hygiene routine at home. The selected strategies included teaching patients and parents and/or caregivers how to brush one’s teeth, identifying the most suitable tool to reinforce motivation in taking care of oral health.

Confidence is achieved both during dental sessions at the clinic and at home, using games, videos and other digital activities personalized for each patient. Exploiting the combination of these techniques, we managed to first approach the simplest medical procedures (sitting in the dental chair, opening the mouth, having the oral cavity checked first only visually and then using tools such as mirror and explorer), and then to integrate increasingly complex instruments and methods.

At the same time, the project explored the use of wearable sensors to assess subjects’ physiological response before and after the dental procedure by applying commercial devices and analyzing, ex-post, the modification of the Autonomic Nervous System activity via signal processing related to electrocardiogram (ECG) and Galvanic Skin Response (GSR), two significant, well-known physiological indicators of psychological stress, which will be better defined in Section 4.

## 2. Method

### 2.1. Sample

The total sample included 59 subjects (45 males; 14 females) aged between 4 and 16 years (mean age = 9.9 years; SD = 5.43) who were sequentially recruited at the Department of Pediatric Odontoiatry of Santa Chiara Hospital in Pisa during the period January 2019–October 2020. Inclusion criteria were a diagnosis of autism spectrum disorder (ASD) according to DSM-5 criteria [1] and pediatric age. The diagnosis was certified by the Italian Mental Health System. All subjects with ASD were native Italian speakers (Table 1).

The study was approved by the local Ethics Committee (Prot. N. 10–06/05/2019 AOUP, Pisa, Italy,) and was in accordance both with the ethical standards of the 1964 Declaration of Helsinki and the Italian Association of Psychology (AIP). The protocol for data acquisition with wearable sensors was approved by the Institutional Ethical Clearance board of CNR.

### 2.2. Intervention

#### 2.2.1. MyDentist

MyDentist was developed as a multidisciplinary experimental approach to dental care and prevention for subjects with ASD, combining ICT, behavioral techniques and a specific clinical protocol designed for ASD. The protocol definition came from the combination of a special dentistry foundation, knowledge and previous experience on assistive technologies for people with ASD, and the crucial support from psychologists and speech therapists offering expert advice in the field of diagnosis of and intervention in ASD. The choice of a multidisciplinary approach derived from previous studies in the literature [24,29] and from a pilot study performed in 2017 [27].

The concepts on which MyDentist has been developed are the following: (i) the high frequency of use of general anesthesia; (ii) the difficulty to face a lack of collaboration when working with ASD patients; (iii) manifestations of anxiety in reaction to the context of the visit; (iv) the need to keep consistency between home oral hygiene and oral care at the clinic. Luckily, some recent experiences in this field have led to the development of new forms of intervention, constantly evolving and mainly focused on psychological approaches where dentists explore the use of different communication channels to enhance collaboration and interaction with children (reinforcement, costumes, play activities) [31,32].

For all these reasons, MyDentist builds on this preliminary literature to explore new forms of patient interaction with the clinical environment and with the medical staff. We propose digital activities to reduce anxiety during the visit, to teach medical procedures and familiarize the patient with the environment. Accessible training is delivered through multimodal activities and games based on AAC, exploiting the visual channel, usually the most effective sense in people with ASD [31]. ICT can facilitate the implementation of AAC, making it easy and comfortable to communicate anyplace, anytime through a mobile device (tablet, smartphone). Moreover, digital AAC (delivered via app) is highly scalable (vast amount of items vs. the paper/plastic version) and customizable, allowing one to add new (personalized) items.

A key feature of the MyDentist approach is the complete personalization of the medical protocol in order to adapt it to the particular needs of each ASD patient, to favor a relationship of trust between the patient and the health professional, and to positively exploit the digital activities.

#### 2.2.2. The MyDentist Web Application

Several studies underline the positive effect of daily use of ICT tools with people with ASD, in both learning contexts and social situations [38,39,40,41]. As reported in these studies, people with ASD seem to like innovative educational approaches enhanced by ICT tools mainly because when using technology, they can avoid typical issues involved in human interaction, such as impatience, feelings of inadequacy, unpredictability of people’s behavior, and poor recognition of emotions, irony, and figurative language.

The use of ICT to facilitate dental care delivered to patients with ASD is a relatively unexplored field. Isong et al. [35] and Barry et al. [42] both investigated the power of visual stimuli on achieving ASD patients ‘cooperation during medical procedures. However, in both these studies ICT tools were not personalized and the children were passive users of digital content. Instead, during the MyDentist experience we tried to offer a personalization of the medical protocol in order to adapt it to the particular needs of each ASD patient, thus making it possible to favor a relationship of trust and to best take advantage of the digital activities proposed directly involving the patient, who become an active actor in the care protocol.

MyDentist delivers a large amount of digital material personalized for each patient: social stories and video models describing all the procedures, the behaviors at the dental clinic and the correct dental care at home, videos and photos taken during dental sessions, and serious games. Managing all this material is very complex and error prone, so MyDentist supports data management with a web application, accessible from any browser on both PCs and tablets. The application helps dentists to build personalized virtual paths following the intervention protocol of a single patient and to provide each patient with all the material needed to boost self-confidence, for instance the photos taken during previous sessions, homework to prepare for the next session and other relaxing and motivating material. The app can be used in many different ways:

- Digital Copybook mode (for all patients): during visits to collect photos and videos (selfies and not) that the child can review and show to family members once at home;
- Mirror mode: during the visits used in camera-selfie mode to allow the child to see himself during dental practices;
- Distractor mode: during the visit to distract or to relax with favorite videos/pictures;
- Reinforcement mode: at the end of the session to perform entertainment and play activities that work both as reinforcement and as consolidation of the activities carried out during the visit;
- Familiarization/learning mode: at home to recall previous visits, to prepare for the next one and to perform digital activities assigned by the dentist (playing games, watching videos, practicing social stories, etc.).

**Figure 1:**
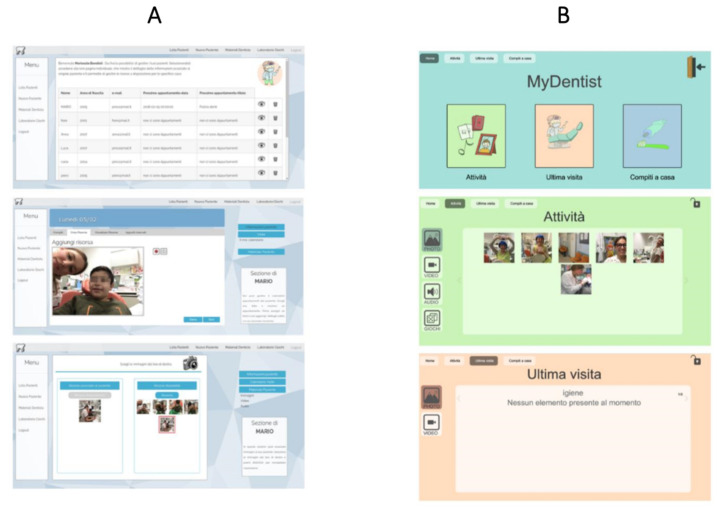
The MyDentist User Interfaces: the dentist’s section (**A**) and the patient’s section (**B**).

The Web Application (Figure 1) consists of two areas:

- Dentist Area: where the dentist creates the personalized profile of each patient, in order to schedule a visit, build a personalized clinical path, collect multimedia materials of visits, create personalized clinical programs by customizing games, share resources and assign home tasks.
- Patient Area: where each patient can access his/her personalized path. This includes games showing dental procedures in an amusing way (e.g., sequences, puzzles, memories); video models for dental procedures (for instance showing how to use a toothbrush), interactive PDF files to get used to dental clinic sounds, social stories that introduce the dental environment, procedures and/or objects and photos/videos collected during visits.

#### 2.2.3. The MyDentist Intervention Protocol

In MyDentist each child follows a personalized path, which starts with one or more familiarization sessions, which goal is to make the child used to the people and the environment. After that, we applied one of three dental procedures. First, prophylaxis with either manual or ultrasonic instruments, which can be challenging for patients with ASD due to the sound of ultrasonic scalers, or noxious tasting prophylaxis paste with manual instruments. Second, fissure and pit sealing, which requires a degree of cooperation, are usually difficult for ASD people. This procedure, despite being a completely atraumatic and not painful, cannot be interrupted, and the patient must always keep their mouth open from beginning to end. In addition, the various materials used have a pronounced taste that may not be easily tolerated.

Third, dental treatment of deciduous and permanent elements is carried out using rotating instruments such as a dental drill and a micromotor. The use of instruments with diamond burrs is reserved for ASD patients with high collaboration skills, since these instruments could damage oral tissues in case of abrupt movements by the patient. In addition, these procedures are often preceded by injection of local anesthesia to cancel painful stimuli. Finally, restoration must be carried out using adhesive materials that require a dry oral setting and therefore a maximum control of salivation.

## 3. Procedure

### 3.1. MyDentist Intervention Path

**Figure 2:**
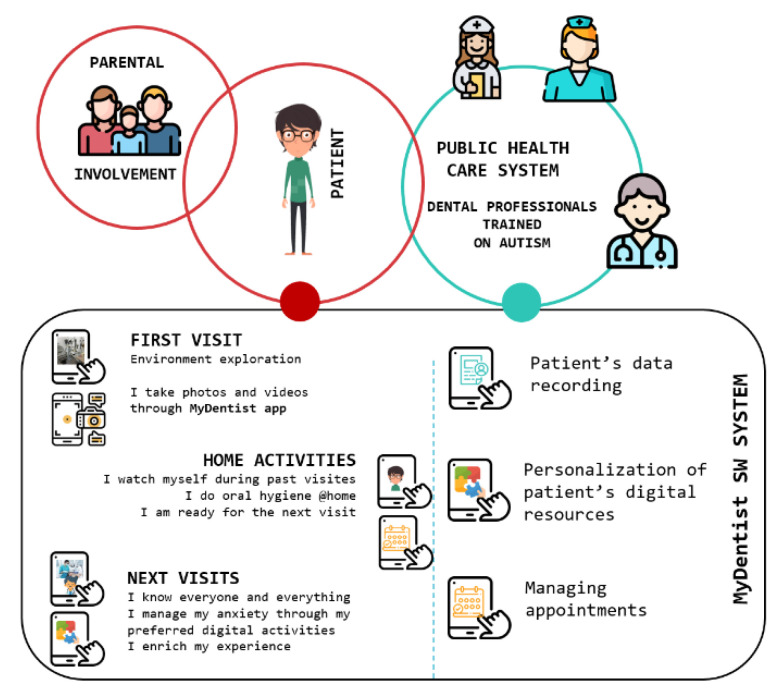
MyDentist global view.

All patients shared a basic path: a preliminary interview with parents, a first visit, some familiarization sessions via a psychological approach, and some operative sessions. A global vision of the MyDentist intervention, common to all the patients, is shown in Figure 2. However, for each step, a personalization of the path of prevention and treatment was defined, according to (1) the specific dental needs (urgent interventions/no urgency, need to improve and increase the norms of oral hygiene at home, need to familiarize the child with the dental clinic in order to be able to carry out preventive interventions); (2) the patient’s needs (especially in relation to the educational/therapeutic paths that he/she was already following), also analyzing any manifestation of the spectrum in relation to the procedures carried out. The personalization path is created cooperating with parents and caregivers and based on observations during the sessions. Visits are basically setting adaptation sessions in which everyone arrives at a state of trust by progressively introducing him/her to the dental team, the clinical environment, the tools used, and the procedures to be implemented. It is fundamental not to force the subject to carry out certain actions but to perform them in their own time and way; it is also important to inform the parents/caregivers so that they also respect the chosen timing to proceed through the path.

#### 3.1.1. Preliminary Interview with Parents

The interview with the parents takes place in the absence of the patient in order to collect as much information as possible about the child. During the interview, the patient’s medical records are filled in with medical, family, and dental history data, which are essential in order to obtain the most complete patient profile. Eating habits (frequency and type of meals and snacks) including the use of sweetened drinks or soft drinks, are also noted. Moreover, oral hygiene habits at home are recorded: type of toothbrush and toothpaste, whether the patient is able to rinse, how many times a day the child brushes his/her teeth, whether alone or with the help of an adult, whether other aids are used (floss, mouthwash, etc.). We also record information about the neuropsychiatric assessment of the child: the diagnosis of ASD (when and by whom it was made, whether there are comorbidities) and the characteristics of the patient’s communication (verbal or non-verbal subject, and if verbal whether structured or not), understanding, use of non-verbal language (e.g., pointing), presence of hyper-sensitivity. Next, we evaluate the potential use of images as communication support (what kind, in what way, with whom they are used), the use of ICT supports, the positive stimuli (e.g., music, cartoons) that can be used, and sports or hobbies if any. Finally, we collect information related to the child’s present and past therapies (when, for how long).

The interview allows us to collect information about how to plan interventions and helps us to better identify the objectives to pursue, which must be tailored to fit the patient’s needs. Finally, we explain to the parents what to do with their children (not to force children to do things they do not want to do during the initial meetings, prepare them with the material needed, communicate with the team periodically in order to record every change); we also instruct the parents on the correct habits to maintain for good oral health.

#### 3.1.2. First Visit

The first visit helped the dentist to get to know each child and to test its reactions to simple requests such as to sit down on the dental chair or to open his/her mouth. During the visit, thanks to the parents’ cooperation, we offered a kit of digital tools to the child to help him/her become familiar with the dentist and the clinical environment, before carrying out the medical intervention. The dentist (helped by the ICT mediator) personalized the kit’s components in all the intervention phases depending on the child’s needs. According to the indications of the parents and therapists, each visit (the first and the other ones) includes the possibility of familiarization at home with the dental activities to be performed in the following sessions through the use of ICT tools such as video modeling, social stories and cognitive learning games (puzzles, memories, sequences), also referring to photos and videos from previous sessions, with the aim of keeping anxiety under control.

#### 3.1.3. Familiarization Session via a Psychological Approach

**Figure 3:**
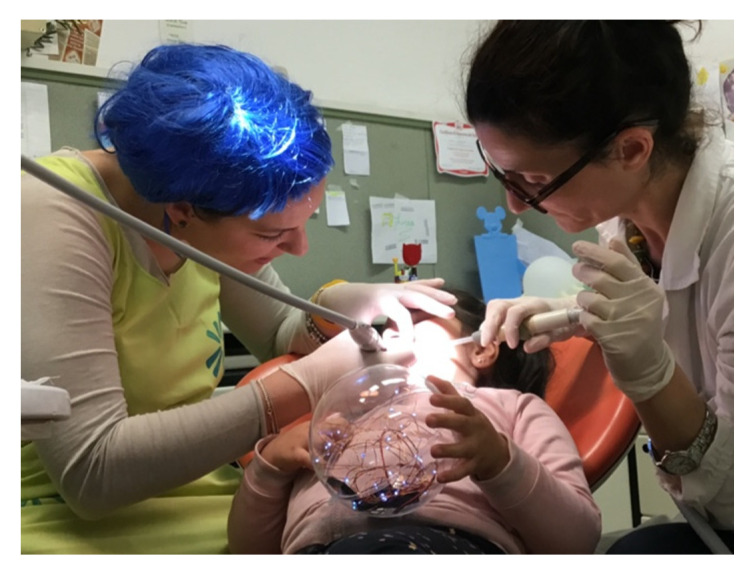
Dental room and the involved staff.

In order to facilitate the collaboration of the child during the visits we exploit the use of technological devices such as the tablet (as detailed below) as well the use of playful-communicative supports such as costumes and disguises (i.e., Snow White, Joy of Inside Out, Alice in Wonderland) and thematic songs (Figure 3).

All the procedures, from the waiting room to the final greetings to the team, are carried out by having the child participate and making him/her aware of what the dentist is doing. This is done by communicating the steps of the operative phase. Usually, we adopted multiple strategies based on the child’s skills: (a) supporting the verbal instructions with visual cues; (b) Augmentative and Alternative Communication (AAC) and video-modeling.

It is also particularly important to use positive reinforcement after reaching a specific objective and at the end of each session.

#### 3.1.4. Operative Sessions

In the case of a familiarization visit (first visit or other visits if needed) the activities are more flexible but aim to keep to basic objectives such as sitting, collaborating, opening one’s mouth, etc. Conversely, in the case of an operative session, such as the treatment of cavities or sealing molars, actions are more focused on small measures that increase attention and reduce distractors.

Whatever the type of visit, the MyDentist approach is based on reshaping the objectives of the session according to the collaboration shown by the patient, even during the visit itself, calibrating compliance with the needs expressed (directly or indirectly) by the child, with the aim of pushing the patient beyond his/her limits. The tablet is considered an integral part of the interaction with the patient but is also an advantage for the staff when collecting material for social stories, cognitive learning games, audio, and video archives.

Only when goals or sub-goals (at least one for each session) are considered achieved, the planned protocol moves to the reinforcement/play phase. Taking care to separate clearly these two stages, especially in the patient’s perception, this final phase is devoted to reward the child with a prize such as listening to his/her favorite songs or watching his/her preferred video, playing or drawing with the tablet. Leveraging on the habits of the patient, this moment represents a particularly important ritual for the success of the visit, facilitating the maintenance of concentration even in the most operational sessions.

## 4. Instruments

### 4.1. Questionnaires

The research team developed the questionnaires to use in this study. Both questionnaires were pre/pilot tested on a subset of 10 parents. Two experts examined the questionnaires for face and content validation. One of the experts has 15 years of experience in the field of Autism Spectrum Disorder. Both experts have a master’s degree in psychology and one of them has a PhD in Developmental Neurosciences. They have been involved in several national and European research projects. For face validity, the experts were asked if all questions were clearly worded and would not be misinterpreted. For content validity, the experts evaluated the relevance of the questions using a scale of 1 to 3, where 1 = not relevant, 2 = relevant but not necessary, and 3 = absolutely necessary. The experts were also asked if other questions should be added to the questions. The remarks of the two experts were collected and discussed and were used to revise the questionnaires. The experts examined the revised questionnaire and declared agreement with its content and clarity.

Parents anonymously completed two questionnaires in order to describe their personal experience with MyDentist. Questionnaire A was aimed at evaluating the approach of children to the dental experience. It was filled out two times, at T0 and after 6 months, T1. It is an 18-item parent-report measure designed to record the behaviors of subjects. Each item describes a specific behavior and the parent is asked to rate its frequency on a four-point Likert scale (0, never; 1, sometimes; 2, often; 3, regularly).

After 6 months of the MyDentist experience, a second questionnaire (Questionnaire B) was sent to the parents of children who participated in the MyDentist project. The questionnaire included 16 items with a positive or negative orientation toward the multi-media support. The questions regarded the evaluation of tablet use and the improvement (if present) in the child’s skills. The parents had to answer through a Likert scale from 0 to 3 (0  =  no, 1  =  little, 2  =  quite, 3  =  very).

### 4.2. Psychophysiological Assessment

As mentioned in the previous sections, aside from observing a positive effect of the MyDentist approach as indicated by the results of the questionnaires, we would like to answer the following question: Is it possible to have an “objective” measure that provides an indicator of the effect of MyDentist on stress?

To answer such questions, our idea is to assess the response of the Autonomic Nervous System (ANS) using wearable devices. The ANS measurement is a useful instrument for evaluating the subject’s stress and emotional state in ASD. Indeed, when perceiving a stressor, self-regulating processes start by automatically activating the ANS, its sympathetic component [43,44]. The ANS can be monitored non-invasively and reliably by means of wearable sensors, shown to be effective and useful even in young children and in children with disabilities [45,46,47].

Therefore, in order to be able to objectively evaluate the influence of the practice proposed with the MyDentist approach in modulating the anxiety state of subjects with ASD who undergo dental treatment, we intend to use non-invasive devices for recording ANS activity. Specifically, the idea is to assess the ANS activity before and after each dental session and to longitudinally monitor each child for an observation period of approximately 4–6 months. With this strategy, it would be possible to observe whether the dental session-associated stress is reduced as time passes thanks to the MyDentist application.

To assess the feasibility of applying such approach, we evaluated the ANS activity using wearable devices before and after the first dental session with MyDentist, with one child participating in the study (5-year-old child with ASD level 2 according to DSM-V criteria and an IQ in the “mild” range).

The child was equipped with two wearable sensors, the first of which for monitoring an electrocardiogram (ECG) and the other one devoted to galvanic skin response (GSR). The ECG signal was acquired through the Shimmer ECG sensor, v.2 (Shimmer Sensing, Dublin, Ireland) attached to a fitness-like chest strap manufactured by Polar Electro Oy (Kempele, Finland), in turn interfaced with the human body through two dry electrodes. The sampling frequency was set to 500 Hz. The GSR signal was captured by the Shimmer3 GSR+ sensor (Shimmer) by adhering to two neighboring fingers of the subject’s non-dominant hand via comfortable rings embedding dry electrodes, with optimal comfort for the subject tested. As for the GSR, the sampling frequency was selected from among the ones available from the sensor firmware, set to 51.2 Hz. The signals were acquired while the subject was seated comfortably on a chair at a table in a quiet room for 5 min both before and after the session.

The acquired physiological signals were processed using Matlab (Mathworks, Natick, MA, USA). The ECG signal was analyzed for the calculation of the tachogram (RR series, i.e., the time elapsed between two successive R-waves), taking advantage of the Pan-Tompkins algorithm [48] and to extract both time- and frequency-domain features, including:

- Heart rate (HR): the number of contractions of the heart occurring per time unit, expressed in bpm;
- Root mean square of successive differences (RMSSD): measure of heart rate variability (HRV), specifically related to parasympathetic activity, expressed in ms;
- Normalized component of the power spectral density of the ECG signal at low frequency (0.04–0.15 Hz) (nLF); it is related both to the sympathetic and parasympathetic response;
- Normalized component of the power spectral density of the ECG spectrum at high frequency (0.15–0.4 Hz) (nHF); it is mainly related to the parasympathetic response;
- Low- vs. high-frequency components of the power spectral density of the ECG spectrum (LF/HF Ratio): under controlled conditions, it expresses the balance between the sympathetic and parasympathetic nervous system branches [49].

GSR signal was analyzed using Ledalab V3.4.9 (General Public License (GNU), Graz, Austria), a Matlab-based tool. The signal was first filtered by a moving average filter (n = 8) for artifact removal, followed by a continuous decomposition analysis (CDA) via the dedicated Ledalab function, leading to the extraction of tonic (and phasic, not used here) components. Indeed, just the tonic phase of the GSR signal was extracted and analyzed, since the main goal of the analysis was related to the assessment of slow changes in the autonomic activity rather than to the response of a single, time-defined stimulation that would have affected the phasic component of the GSR, as well [50].

## 5. Descriptive Analysis

Given the exploratory nature of the present study, there was no assumption of the sample size. Categorical data were summarized into frequency counts, percentages, and contingency tables, which were analyzed using chi-square analysis for both questionnaires. Analyses were carried out using SPSS version 21.0 for Windows (SPSS Inc. Chicago, IL, USA).

## 6. Results

### 6.1. Questionnaires

Significant results were shown from T0 (before initiating MyDentist) to T1 (after 6 months of MD experience) in 14 of the 18 questions to which the parent had replied (Table 2).

Families have positively assessed the use of multi-media support in MyDentist. Parents acknowledged that the multi-media supports were appropriately customized, facilitated a good compliance of the child with the dental setting and reduced the child’s stress during the dental examination. According to parents’ answers, the use of multi-media supports made children more familiar with dental hygiene, more adapted to and cooperative with the dental setting. Moreover, sensory issues and problematic behaviors appeared more manageable for the children. The multi-media supports helped children in translating acquired skills also out of the dental clinic and in learning the autonomous use of tools for dental hygiene. Parents evaluated the MyDentist project as useful, considered the advice received during the MyDentist experience to be applicable at home and recommended MyDentist to other parents of children with ASD. For only 1/3 of parents the multi-media support was considered hyper-stimulating (Table 3).

### 6.2. Psychophysiological Assessment

**Figure 4:**
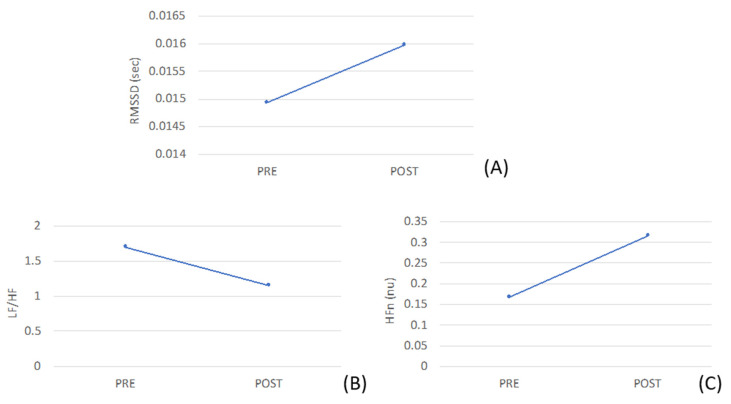
Trend of the features extracted from the R-R series: (**A**) Root Mean Square of the Successive Differences of the R-R signal (RMSSD), (**B**) LF/HF ratio, (**C**) normalized High Frequency component of the ECG signal.

**Figure 5:**
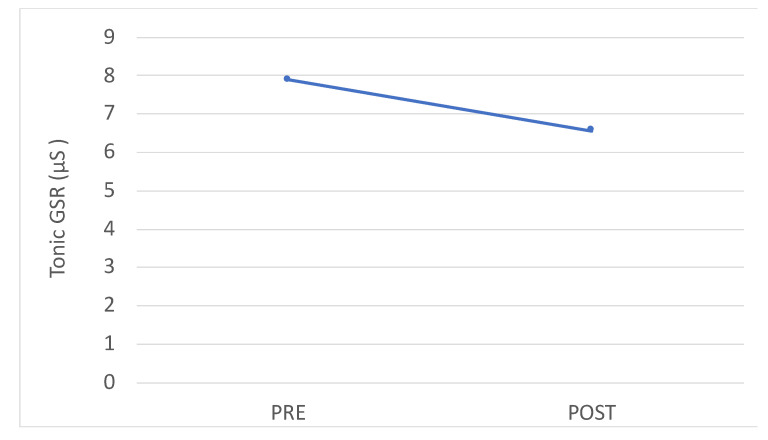
Trend of the tonic component of the Galvanic Skin Response (GSR) signal.

Concerning the assessment through wearable sensors, in Figure 4 it can be observed that after the dental session, the parasympathetic contribution increased, as suggested by the rise in RMSSD, nHF and by the decrease of LF/HF. Accordingly, the tonic component of the GSR signal decreased with respect to the baseline pre-session after the dental session, as observed in Figure 5.

## 7. Discussion

Children with ASD have poor oral health and they present high care demands, which requires much time, effort, and patience [8,51]. Findings of this study suggest the feasibility of the MyDentist approach and the positive role of technology support [52]. Most of the families of the children involved in this study showed a positive level of involvement (there were no dropouts). Our descriptive data suggest that the children learned the basis of adequate oral care following the rules for proper dental hygiene, using a toothbrush and brushing teeth after meals. Moreover, they showed progressive compliance with the dental visits remaining in a seated position and managing their sensory issues better. Parents and caregivers alike manifested satisfaction for the MyDentist protocol and they strongly recommended it to parents of children with ASD. The ICT use in the MyDentist protocol was positively accepted by the families involved. They considered that the multimedia supports were appropriately customized to meet the child’s needs; it facilitated good compliance of the child with the dental setting and reduced the child’s stress during the dental examination. ICT has the potential to offer ASD users increased rehabilitation and support to empower their abilities, anytime, anyplace, and on any device (https://iite.unesco.org). Repetitive and predictable answers could help these patients control anxiety and accept new medical practices. Thus, to increase positive results, personalization becomes crucial for making the surrounding environment comfortable and user-friendly.

Our findings also underlined the importance of ICT as an important tool for teaching children with ASD better cooperative behaviors aiding them in the familiarization process with rules and techniques of appropriate oral hygiene. It is well-recognized that children with ASD experience challenges with communication which can negatively interfere with professional oral care [52]. However, most children with ASD tend to process visual information more efficiently than auditory information [53]. For these reasons, in our study AAC and video-modeling as teaching supports were used. Another key finding of the application of the MyDentist protocol is the improved collaborative behavior of the child in home oral hygiene practices. This suggests the potential translational effect of the MyDentist experience.

In addition, the preliminary investigation in a single subject regarding the use of wearable sensors to assess autonomic nervous system response suggested the feasibility of using this approach to evaluate the child’s physiological response, thus confirming our previous studies [41,42,43]. This approach allows us to obtain useful information about the anxious response to a potentially stressful event [36]. In this specific child and for this specific session, it seemed that the child was more comfortable and relaxed after the session, expressing an increase in parasympathetic activity. It would be interesting to observe whether after using the MyDentist approach regularly, the child is more relaxed also before the session. In the near future, we intend to include a fairly large group of subjects compatibly with their profile in order provide more information about the response of the child than that merely obtained from behavioral observation. This would be extremely important for objectivizing the effect of the intervention and implementing more effective and individualized approaches.

In order to evaluate the cost for a generalization in other settings, we need to highlight that the MyDentist intervention differs from the regular dental care intervention for people with ASD in these aspects:

1. Professionals are trained in how to treat people with ASD. As mentioned in the Introduction, to increase the probability of successful dental treatment for patients with ASD, dentists should have an in-depth interdisciplinary understanding of ASD in terms of symptomatology and functional profiles, but unfortunately the professional training offered by most university programs does not deal specifically with people with ASD.
2. Professionals are also trained to use the MyDentist application, which helps to set up a personalized use of the ICT tools
3. In the first period of the intervention, an additional person acting as an ICT-mediator for both professionals and patients/caregivers joined the dental clinic staff.

Indeed, the cost of the MyDentist intervention can be estimated in:

- Cost for the design and development of the MyDentist application;
- Cost of the cloud-storage service;
- Cost of a tablet for the dental clinic;
- A 2-year employment contract for an ICT mediator.

Considering our experience, the professionals were already trained in how to interact with people with ASD and how to use the MyDentist application, since they participated in its design. Patients at home used their own mobile devices.

The estimated cost for replicating the MyDentist intervention in other settings should consider:

- For professionals:
  - Training regarding people with ASD and the most appropriate way to interact with them;
  - Training to use the MyDentist application: to this aim, we are finalizing all the documentation such as user manual, tutorials and demo, which will be freely available.
- For the clinic:
  - A 6- or 12-month employment contract for an ICT mediator.

The MyDentist application will be available for free, at no cost.

Regarding the training of dental staff, it depends on several factors such as the type of course, the duration and the organization that provides the course. However, in order to indicate in more detail what the cost may be, we believe it may be sufficient a brief training course with an ASD expert (e.g., psychologist, neuropsychologist or speech therapist). Furthermore, dental staff will also need an additional day for autonomously training on the MyDentist application reading the documentation and trying the app.

The ICT mediator is a person with skills on the use of digital tools in order to support the work of the dental staff showing how and when to use the application and the tablet. This does not require a very high technical ICT skills so a graduate person may be sufficient. However, the ICT mediator must also have ASD training, together with the dental staff, in order to know how to approach and interact with people with ASD.

In some cases, the treatment path may be longer than estimated, but there are benefits for both the patient, who could be treated while safely avoiding sedation for minor interventions, and presumably for the health system, due to the increase in successful interventions and the decrease in missed appointments or incomplete treatments caused by a lack of patient collaboration (increasing efficiency and reducing unnecessary costs).

## 8. Limits of the Study

In this preliminary study, some limitations must be acknowledged. The most relevant is undoubtedly the constrained dataset that strongly limits wide-ranging conclusions. In order to overcome the limits of our study, future investigations should consider: (1) a more systematic data about functional profiles of each experimental participant (ADOS/IQ scores; and robust characterization of the sample in terms of sensory sensitivity and co-morbid conditions; (2) the inclusion of a control group with ASD (e.g., assignment to routine/usual treatment) with a randomized assignment to conditions or, possibly, the adoption of a crossover strategy over the entire population of volunteers; (3) to collect pre- and post-intervention data on parents stress’ degree; (4) the lack of a protocol or decision-making tree for the use of the MyDentist intervention.

Finally, a limit of our study was the evaluation of the autonomic nervous system response in only one child for a single session.

Thus, further research is needed to confirm our interesting preliminary (and descriptive) data to determine the effectiveness of the intervention. Moreover, in an upcoming study the enrollment of a larger number of subjects will be important in order to create subgroups of more homogeneous samples in terms of age range, IQ range and oral hygiene issues.

## 9. Future Direction

Given the overall encouraging results achieved by the exploratory study presented herein, we expect to extend the recording of physiological parameters to the visit phase, at least in a subgroup of individuals who might be more compliant with being equipped with wearables sensors, even during the visit, by the dentist. Such individuals, selected from among the subjects with high-functioning ASD, will compose the cohort for this further evaluation, and will be followed in the next study. This would enable comparing the compliance and the psychophysiological response, and therefore the overall benefit brought by the MyDentist App, with respect to the previous literature dealing with dental research, stress and ASD [54,55,56,57]. Finally, other parameters could be extracted from the physiological signals, including frequency of non-specific skin conductance responses (NS-SCRs) for the GSR.

## 10. Conclusions

Networking between families, caregivers and health providers should be activated with the aim of making everyone aware of the dental health problems that children with autism have to face [13,58,59].

It is essential to combine the efforts and professionalism of all the figures involved in helping a child with autism in order to draw up ad hoc guidelines for dental health in children with ASD.

In conclusion, these preliminary findings regarding the MyDentist project have shown the feasibility of dental care in children with ASD. Furthermore, the project showed the parents’ acceptance of the MyDentist project and the feasibility of dental care in a public service without additional costs for families. In the first phase of the dental care program, an additional cost is required for dental clinics facilities to teach clinicians how to implement the intervention with persons with ASD, and to proficiently learn how to exploit the MyDentist program specificities. Moreover, an ICT mediator could be essential to provide the necessary technological support. However, also considering these constraints, we believe that the cost effectiveness is largely in favor of the intervention. Potential benefits for these special-needs patients are extremely important both in term of health (through prevention or treatment, if needed) and in terms of general wellbeing.

## Figures and Tables

**Table 1:** Socio-demographic characteristics and functional profile of the sample.

Age	(in Years)
Mean	9.9
SD	5.43
**Gender**	*n*
Males	45
Females	14
**DSM-5–ASD Severity Levels**	
Level 1	5
Level 2	37
Level 3	17
**Cognitive Functioning**	
Normal range	5
Mild	4
Moderate	28
Severe	19
**Language**	
No words	29
2–3 words	14
Fluent	16

**Table 2:** Chi Square results between T0 and T1 at Questionnaire A.

	Never (0)		Sometimes (1)		Often (2)		Regularly (3)		Chi Square
	t0	t1	t0	t1	t0	t1	t0	t1	χ^2^; *p*
	n	n	n	n	n	n	n	n	
Q.1 Does your child like to brush his teeth?	12	8	33	27	9	12	5	11	4.07; ns
Q.2 Is it possible to teach oral hygiene?	9	8	34	8	11	34	5	8	28.59; <0.001
Q.3 Is your child able to follow the rules for proper dental hygiene?	15	1	28	8	6	26	10	25	42.28; <0.001
Q.4 Is your child capable of using a toothbrush?	27	8	20	34	5	8	3	8	16.84; 0.001
Q.5 Is your child capable of putting toothpaste on the toothbrush?	32	17	16	8	6	17	5	17	19.06; <0.001
Q.6 Does the child use dental floss?	55	55	2	2	1	1	1	1	0; ns
Q.7 Does the child brush his teeth independently?	32	8	19	25	6	24	2	1	26.34; <0.001
Q.8 Is your child able to brush his teeth independently?	8	1	1	8	8	39	38	8	50.89; <0.001
Q.9 Is your child capable of brushing his teeth properly?	13	8	21	17	12	8	13	25	6.19; ns
Q.10 Is your child able to brush his teeth twice a day after meals?	13	25	26	8	11	24	9	1	24.54; <0.001
Q.11 Does the child brush his teeth after meals?	23	8	15	17	9	8	12	25	12; 0.007
Q.12 Does the child experience hypersensitivity (i.e., toothbrush)?	25	42	20	12	8	3	6	2	10.58; 0.014
Q.13 Has the child ever had dental treatment?	48	29	1	17	6	2	4	10	23.47; <0.001
Q.14 Has it ever been necessary to resort to general sedation?	29	1	9	1	5	1	15	56	58.87; <0.001
Q.15 Does the child have a dental visit at least once a year?	24	55	15	2	9	1	10	1	35.86; <0.001
Q.16 Does your child become upset when he has to go to the dentist?	30	1	20	7	7	25	2	25	63.10; <0.001
Q.17 Is your child collaborative during dental visits?	17	17	13	25	17	8	12	8	7.82; 0.049
Q.18 Does he have difficulty remaining seated for the duration of the visit?	12	9	33	29	9	11	5	9	2.02; ns

The result was significant at *p* < 0.05.

**Table 3:** Percentage of parents’ answers to Questionnaire B.

	Questions	No	Little	Quite	Very
		%	%	%	%
Q.1	Was the multimedia support used in MyDentist (MD) useful?	0	0	40	60
Q.2	Was the multimedia support appropriately customized?	0	0	37.9	62.1
Q.3	Was the experience of MD appreciated by your child?	0	0	33.3	66.7
Q.4	Do you think that the tablet was hyper-stimulating for your child?	38.7	19.4	9.7	32.3
Q.5	Did the use of the tablet facilitate good compliance of the child with the dental setting?	0	0	35.5	64.6
Q.6	Did the use of the tablet reduce the child’s stress during the dental examination?	14.2	0	42.9	42.9
Q.7	Did the child also use the tablet to become familiar with dental hygiene?	3.2	22.6	51.6	22.6
Q.8	Have you found the child to be better adapted to the dental setting?	0	0	71	29
Q.9	Is the child more cooperative during the dental examination?	0	6.2	25	68.8
Q.10	Is the child more able to control sensory issues during the dental examination?	0	0	48.9	51.6
Q.11	If present, have problematic behaviors during the dental examination decreased?	7.7	13.3	36.7	43.3
Q.12	Is the child more collaborative in performing home hygiene?	0	11.7	50	38.3
Q.13	Is the child more autonomous in the use of the suggested tools for dental hygiene?	9.4	15.6	46.9	28.1
Q.14	Was the MD project useful?	0	0	10.3	89.7
Q.15	Can you apply at home any advice received during the MD experience?	0	0	69	31
Q.16	Would you recommend MD to parents of children with autism?	0	0	0	100
